# CSF or middle ear effusion? Diagnostical dilemmas in a patient with temporal bone meningioma: A case report

**DOI:** 10.1016/j.heliyon.2024.e28059

**Published:** 2024-03-16

**Authors:** Glen J.F. Kemps, Douwe de Boer, Maud P.M. Tijssen, Dirk H.P.M. Kunst, Jérôme J. Waterval

**Affiliations:** a Department of Otolaryngology, Isala Hospital, Zwolle, the Netherlands; b Central Diagnostic Laboratory, Maastricht University Medical Centre, Maastricht, the Netherlands; c Department of Radiology, Maastricht University Medical Centre, Maastricht, the Netherlands; d Department of Otorhinolaryngology, Radboud University Medical Centre, Nijmegen, the Netherlands; e Department of Otorhinolaryngology, Maastricht University Medical Centre, Maastricht, the Netherlands

**Keywords:** Cerebrospinal fluid, Beta-2 transferrin, Beta-trace protein, Intraosseous meningioma

## Abstract

**Introduction:**

Cerebrospinal fluid (CSF) fistulas are a rare phenomenon, that can lead to life-threatening complications if left untreated. Presenting as rhinorrhea or otorrhea, they can be difficult to diagnose due to admixture of other bodily fluids. Typically, CSF fistulas develop after trauma, but in rare instances, they can be diagnosed in patients with a neoplastic lesion.

**Objective:**

To discuss several steps in diagnosing CSF fistulas.

**Patient:**

A fifty-year-old female with an intra-osseous temporal bone meningioma.

**Interventions:**

For diagnosing CSF admixture in fluids, two tests are looked into: beta-2 transferrin (β2T) and beta-trace protein (βTP) testing.

**Conclusion:**

Testing for βTP is a highly sensitive, quick and non-invasive method to assess CSF admixture in middle ear effusion. Because of its lower cost, faster results and easy sample collection, βTP testing has in our clinic replaced β2T testing. The current case illustrates a rare etiology of a CSF fistula, where β2T testing presumably showed false-negative results and βTP testing showed true-positive results.

## Highlights

• Intra-osseous meningiomas, when found in the temporal bone, can cause cerebrospinal fluid (CSF) fistulas.
• Beta-trace protein (βTP) is the most abundantly found protein in CSF after albumin.
• βTP testing has several advantages over beta-2 transferrin (β2T) testing, such as lower cost, higher speed and easier collection.
• If a fistula is not found intra-operatively, and CSF admixture in otorrhea is still probable, then a subtotal petrosectomy is indicated.

## 1 Introduction

Cerebrospinal fluid (CSF) fistulas pose a serious risk to a patient’s health due to its potential for severe complications, such as bacterial meningitis or intracranial abscesses [[1], [2], [3], [4], [5], [6], [7]]. An important step in managing these fistulas is an accurate diagnosis, with reliable detection of CSF within secretions [2]. There are many aetiologies causing CSF fistulas, the most common being traumatic fractures of the skull base and iatrogenic defects [2,3,5,7,8]. However, rare cases, in which fistulas were caused by intracranial neoplasms have been described [9]. This report presents a patient with a presumed CSF fistula brought about by an intra-osseous temporal bone meningioma.

Meningiomas are intracranial tumours, thought to arise from arachnoidal cap cells, usually affecting adults with a predilection for women in their fourth decade [[10], [11], [12]]. Meningiomas represent 13–19% of intracranial tumours and approximately 1% of all intracranial meningiomas are primary intra-osseous meningiomas [[10], [11], [12]]. In the current report, several diagnostic steps are emphasised, highlighting the challenges of CSF fistula detection.

## 2 Case report

**Fig. 1:**
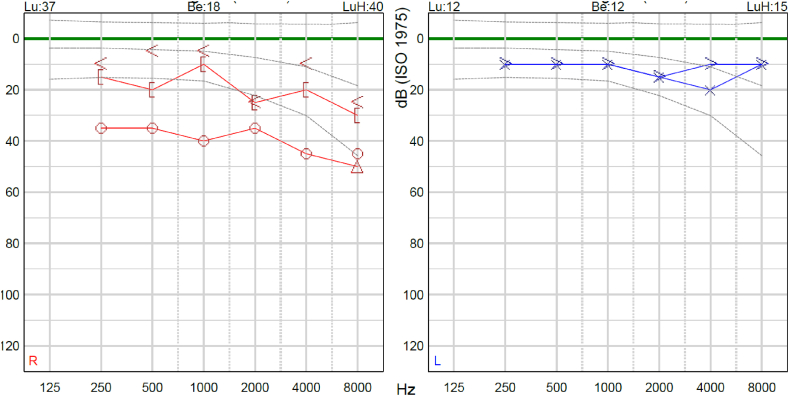
Pure tone audiometry showing right-side conductive hearing loss. Lu = Air conduction threshold (dB). Be = Bone conduction threshold (dB). LuH = Air conduction high Fletcher index (dB).

**Fig. 2:**
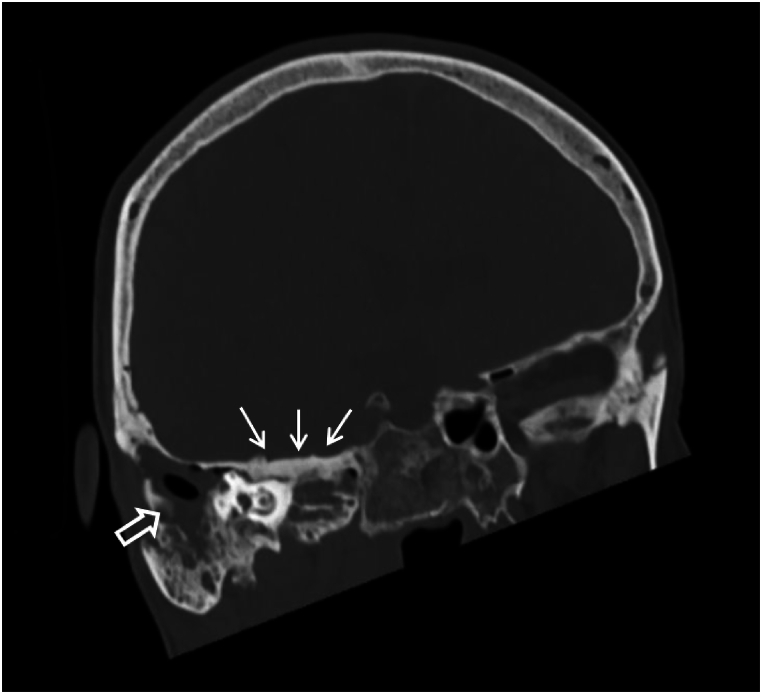
Coronal oblique reconstructed CT slide showing hyperostosis of the right temporal bone (arrows) as part of the intra-osseous meningeoma. There is a postoperative state with an opacified mastoid cavity (open arrow) and intact tegmen tympani.

**Fig. 3:**
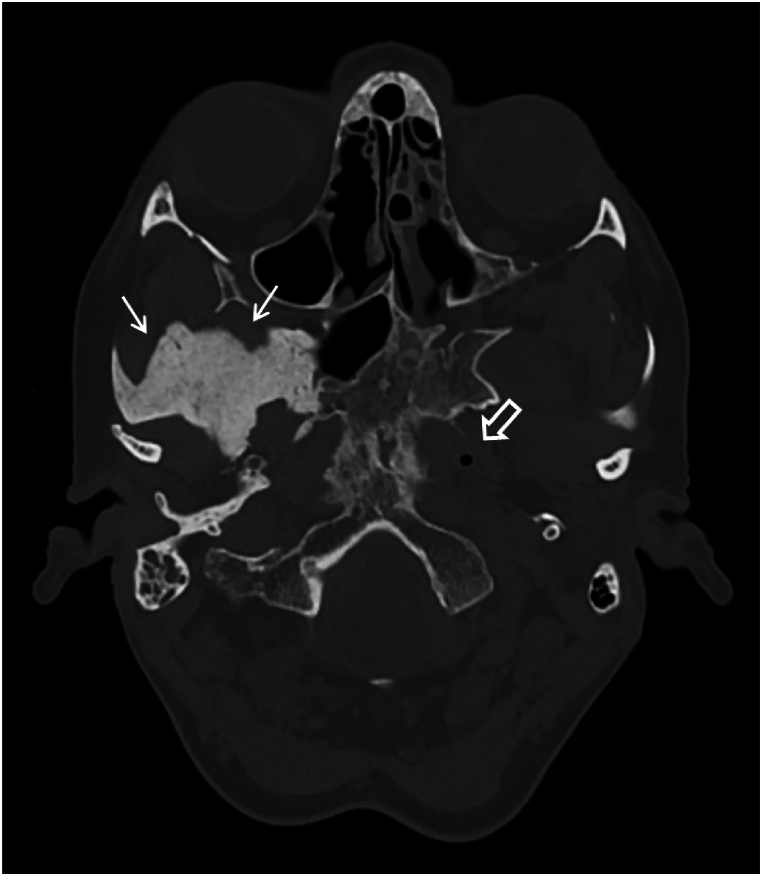
Axial CT image showing evident right temporal bone hyperostosis as part of the intra-osseous meningioma (arrows). Air in the open Eustachian tube on the left (open arrow) and no air in the closed one on the right.

**Fig. 4:**
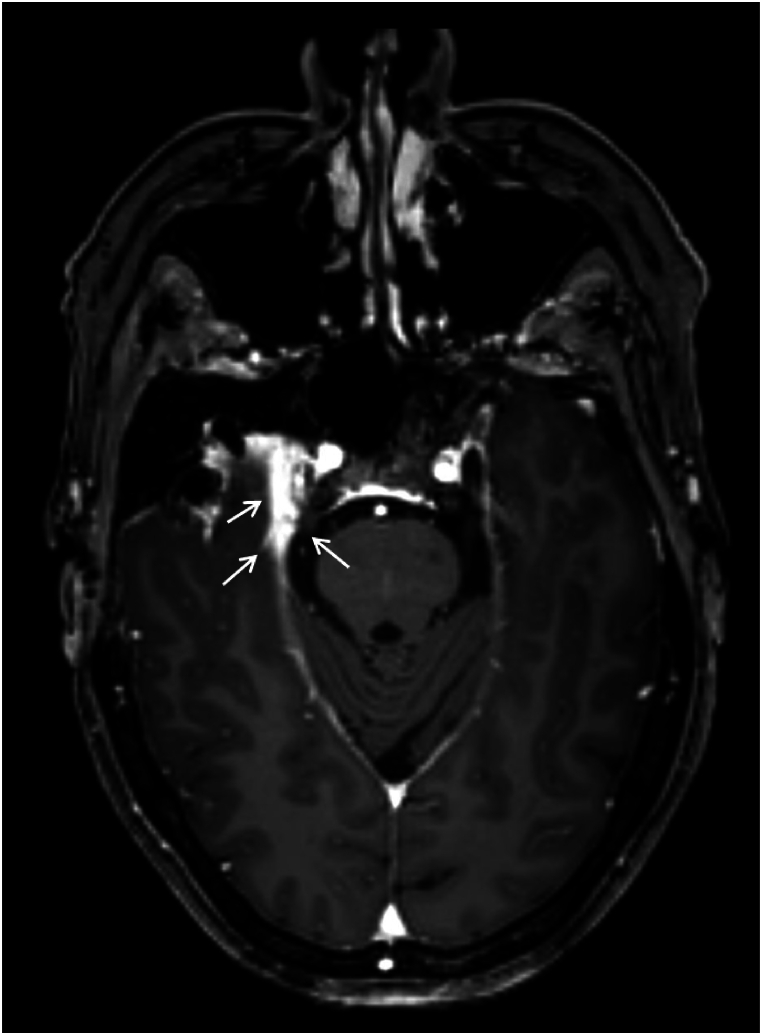
Axial T1 post-gadolinium image. Right-sided temporal hyperostosis is visible. Adjacent to the hyperostosis there is dural thickening (dural tail, arrows) extending to the right tentorium.

A fifty-year-old woman presents with right-sided aural fullness, tinnitus and hearing loss. In her medical history, she is known to have a stable intra-osseous meningioma in her right temporal bone. She had previously undergone surgery in the form of a transmastoidal leak repair of a what was initially deemed spontaneous cerebrospinal fluid (CSF) fistula. Further details or operation notes of this previous surgery were unavailable. Histopathological examination of tissue gathered during that procedure had diagnosed the intra-osseous meningioma. At present otoscopic examination, yellowish fluid was seen behind the right intact eardrum. Otherwise, no abnormal signs were found. Audiometry showed a conductive hearing loss of 40 dB (Fig. 1). CT and MRI imaging were performed (Fig. 2, Fig. 3, Fig. 4). Differential diagnoses comprised either middle ear effusion due to the meningioma obstructing the patient’s Eustachian tube, recurrent CSF leakage, or both.

**Fig. 5:**
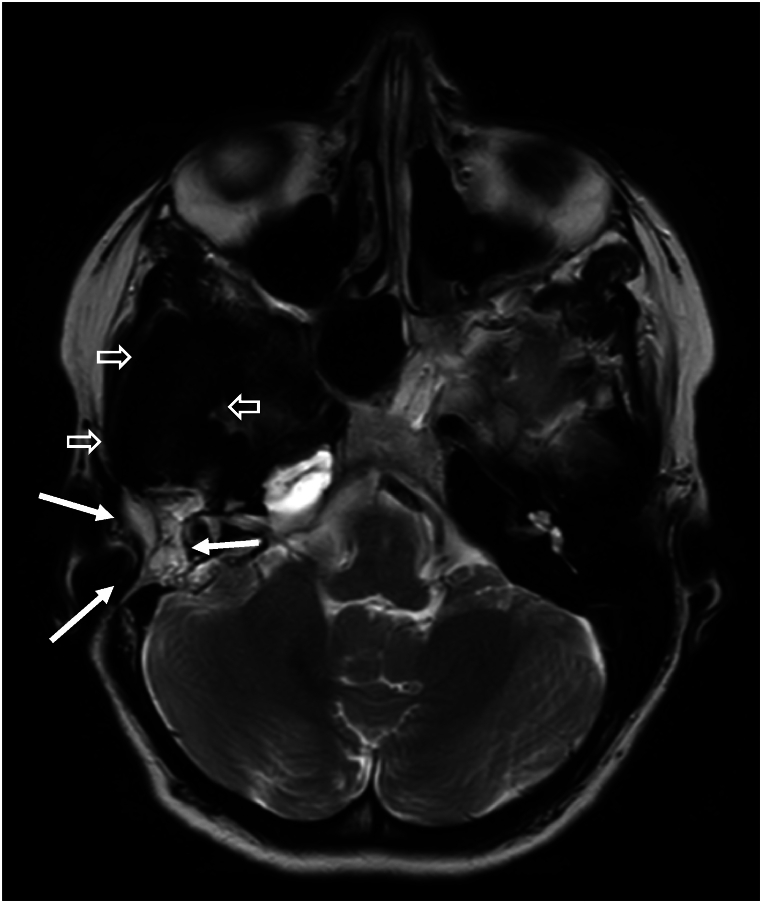
Axial T2-weighted imaging. Hyperintense (fatty) tissue visible after subtotal petrosectomy (white arrows). Persistent hyperostosis of the sphenoid bone anterior of the petrosectomy (hypo-intense signal, open arrows).

**Fig. 6:**
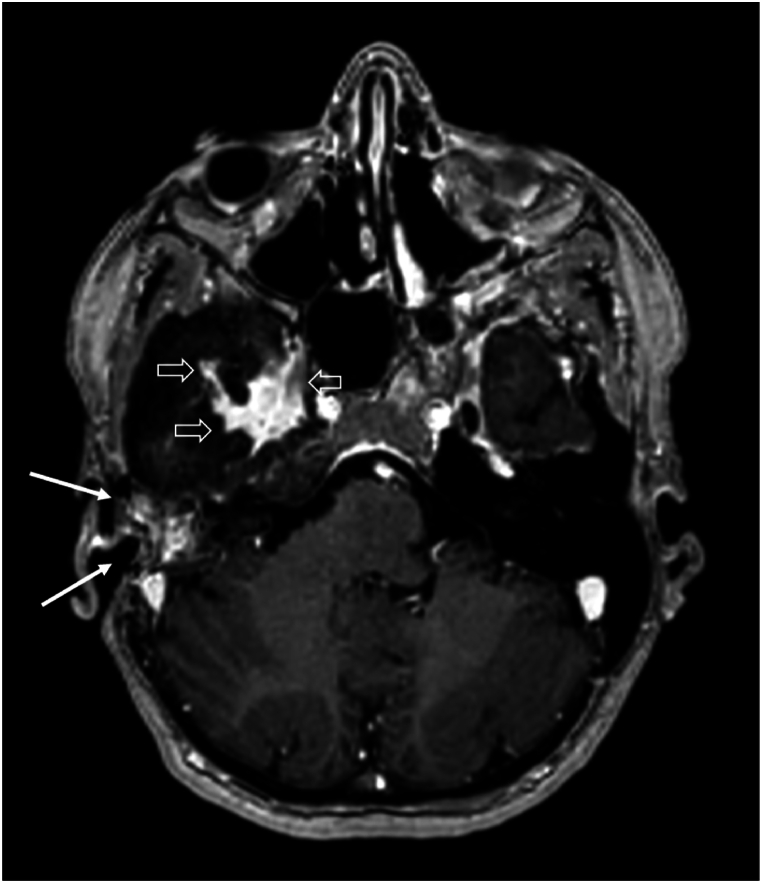
T1-weighted axial reconstruction after gadolinium. Hyperintense (fatty) signal after subtotal petrosectomy (white arrows). Hyperostosis of the sphenoid bone (hypo-intens signal) with solid enhancement at the middle posterior fossa, consistent with residual meningioma (open arrows).

**Fig. 7:**
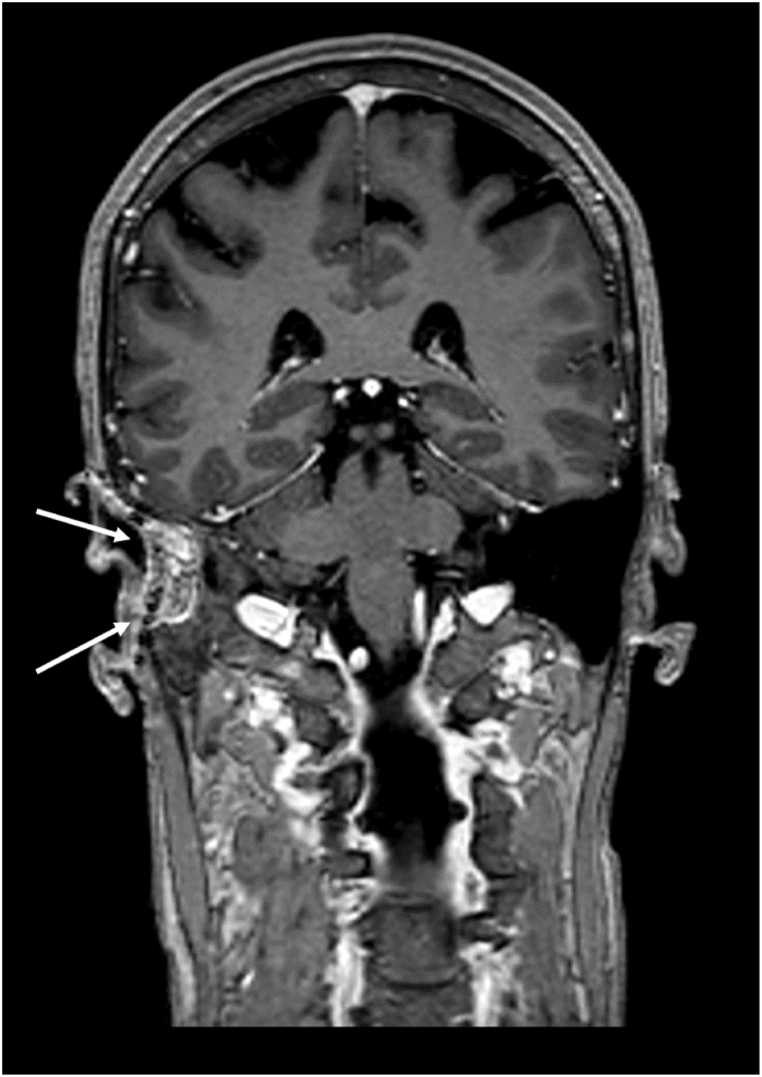
Coronal reconstruction of T1-weighted imaging after gadolinium. The residual meningioma is not visible. Hyperintense (fatty) tissue after subtotal petrosectomy on the right side (white arrows). On the left side normal aspect of air containing temporal bone and middle ear.

A grommet was placed, causing the patient to have continuous otorrhea and infections, which did not respond to topical or systemic antibiotics and which only subsided after grommet removal. Beta-2 transferrin (β2T) testing was performed on the middle ear fluid three times, with negative outcomes. After the patient presented with a Halo-sign, a clinical symptom suggestive of CSF leakage [13], a false-negative β2T test was suspected. Next, the middle ear fluid was tested for beta-trace protein (βTP), the outcome of which was subsequently compared with her serum βTP concentration, according to the algorithm of Bernasconi et al. [6] The patient’s βTP concentration of the patient’s middle ear fluid was 3 mg/L, six times higher than her serum βTP concentration, indicating a likely CSF fistula. After this confirmation, the patient was planned for revision surgery to find and close the leak. Intraoperatively, a pre-existing canal wall up mastoidectomy cavity was found, without any discernible signs of the previous repair. The leak’s suspected origin was through perilabyrinthine and hypocochlear cell tracts, as these were the most suggestive culprits on imaging. Despite the fact that no leakage was seen during surgery, these tracts were closed with temporal fascia, bone dust and fibrin glue. Postoperative recovery was unremarkable. Unfortunately, shortly after surgery, symptoms of middle ear fluid recurred and βTP testing was again found to be positive. Further diagnostic possibilities, such as cisternography and radioactive tracer administration, including its risks and limitations, and therapeutic options, such as attempting to surgically patch the leak either through a transmastoidal or middle fossa approach, were thoroughly discussed with the patient. Complete removal of the meningioma with subsequent reconstruction was also considered. However, due to the extent of the laesion towards the infratemporal fossa anteriorly and towards the tentorium medially, this was not deemed possible without a high risk of complications. Moreover, her meningioma had been stable over the years. Eventually, another attempt was made to find the leak with intrathecal fluorescein. Again, no leakage was seen intraoperatively, after which it was decided to perform a subtotal petrosectomy with blind sac closure at the same stage, as was discussed with the patient beforehand should this scenario occur. The patient’s post-operative recovery was uneventful. Her bone-conduction threshold on audiometry were found to be the same as preoperatively and she opted for a bone conductive hearing aid under local anaesthesia at a later stage. At the time of writing, the patient has been followed-up for two years and 6 months and her meningioma was seen to be stable (Fig. 5, Fig. 6, Fig. 7).

## 3 Discussion

A case is presented with an intra-osseous temporal bone meningioma, which is considered extremely rare, with only a handful of cases found in literature [11,14]. These meningiomas are usually slow-growing and benign, although malignant subtypes have been reported [14]. Similar to the current patient, malignant cases mostly displayed an osteoblastic image on CT scans, but osteolytic types have also been seen [10,11,14]. A differential diagnosis of an osteoblastic meningioma is an osteoma.For osteolytic lesions, this could be fibrous dysplasia, myeloma and metastatic carcinoma [11,12,14]. Due to the proliferative and/or destructive nature of these laesions, CSF fistulas have been seen to occur [9]. Leakage through such fistulas should be reliably confirmed or excluded to avoid potentially life-threatening complications like bacterial meningitis, if left untreated [[1], [2], [3], [4],^6^,^7^]. In literature, an incidence of meningitis of approximately 10% has been reported in patients with CSF fistulas [15]. Within 10 years after an untreated CSF leak, the cumulative risk of developing bacterial meningitis is higher than 85%, with a mortality of 4.1% [8]. Most commonly in clinical practice, the presence of CSF in fluids is determined through the detection of either β2T or βTP [2,4,6,16,17]. Other methods, such as glucose testing or cisternography have been found to be relatively insensitive and unspecific [4,6,16].

β2T is a desialated isoform of transferrin and is found exclusively in CSF and perilymph, making it an obvious choice for CSF detection [4,6,16]. Most widespread β2T tests have high sensitivity and specificity of 94–100% and 98–100%, respectively [3,4,7,16]. However, these tests are done using immunofixation electrophoresis, which is time-consuming due to its need for manual processing that can take hours to days. It also requires experienced laboratory personnel for correct interpretation and is relatively expensive [2,4,7,16,18,19]. In addition, conditions such as liver cirrhosis, kidney disease, alcoholism and genetic transferrin variants might affect test outcome [3,4].

βTP originates in the meninges and the choroid plexus and is present in CSF, perilymph, serum, urine, amniotic fluid and seminal fluid [2]. After albumin, βTP is the most abundantly found protein in CSF. It is involved in numerous functions, such as sleep induction, recovery from seizures and synaptic transmission [2,7]. although βTP is found in CSF in high concentration, its concentration in serum is low [3,16,17], and it has been reported to have the highest CSF/serum ratio of all CSF specific proteins [20]. βTP testing is done with immune-electrophoresis or laser-nephelometry; an automatic, fast (15–20 minutes) and inexpensive process [4,7]. It utilises polyclonal antibodies from rabbits against human βTP, which form an antigen-antibody complex. Light then scatters differently through samples with different amounts of these complexes, allowing even small fraction differences of βTP to be measured. The use of nephelometers to measure plasma proteins is considered routine by biochemists [2].

In an earlier study by Bernasconi et al. an algorithm was devised to interpret different βTP concentrations [6]. Others authors have used either an absolute cut-off value of βTP or a comparison with CSF and serum concentrations [6,18]. Bernasconi et al.‘s algorithm combines both these methods, establishing an excellent sensitivity and specificity of 98.3% and 96%, respectively. The algorithm states that a βTP concentration above 1.3 mg/L in a secretion is suggestive of CSF presence, whereas a concentration below 0.7 mg/L means CSF is absent. When concentrations are found to be in the grey zone between 0.7 and 1.3 mg/L, they are compared with the concentration in the patient’s serum, and if it is equal to or more than twice as high, CSF admixture is likely [6]. Analogous to the use of βTP to detect CSF in nasal or ear secretions, it has been used to detect CSF in pleural effusions and ascites. However, this has been found to be unspecific [17]. Importantly, βTP concentrations are considerably affected by conditions such as bacterial meningitis, which causes its concentrations in CSF to decrease, and renal dysfunction, which causes its serum concentrations to increase [2,7].

Additionally, the method in which a secretion sample is collected has been found to affect the way it can be examined. For instance, secretion collected with polyvinyl acetate (PVA) foam packing (e.g. Merocel/Xomed or Ivalon surgical products) can be tested for βTP, as no distortion has been seen to occur. β2T test results, however, do appear to be influenced by this material, because of its high protein adsorption [2].

In the current case, although there was evident clinical suspicion of CSF leakage, β2T remained negative repeatedly. Positive βTP tests seemed to confirm the authors' suspicions, but then intraoperatively, no CSF leakage was found, despite intrathecal fluorescein administration. It could be reasoned that the patient’s middle ear was filled with effusion because of Eustachian tube blockage by the meningioma or its subsequent hyperostosis, but this would not explain a positive βTP test. In literature, βTP has been found to be expressed in meningioma cells [21]. Therefore, one hypothesis might be that the patient’s intraosseous meningioma itself caused a positive βTP result as opposed to CSF. The means to find out if the patient’s meningioma expressed βTP by testing a histology sample, however, were not available to our institution’s pathology laboratory.

## 4 Conclusion

A patient with intra-osseous temporal bone meningioma and chronic otorrhea should be suspected of having a CSF fistula. Testing for βTP is a highly sensitive, quick and non-invasive method to assess CSF admixture in middle ear effusion. Because of its lower cost, faster results and easy sample collection, βTP testing has in our clinic replaced β2T testing. The current case illustrates a rare etiology of a CSF fistula, where β2T testing presumably showed false-negative results and βTP testing showed true-positive results.

## Funding

No funding sources.

## Ethical approval

Patient’s consent acquired.

## CRediT authorship contribution statement

**Glen J.F. Kemps:** Conceptualization, Data curation, Methodology. **Douwe de Boer:** Supervision. **Maud P.M. Tijssen:** Supervision. **Dirk H.P.M. Kunst:** Supervision, Writing – review & editing. **Jérôme J. Waterval:** Supervision, Writing – review & editing.

## Declaration of competing interest

The authors declare that they have no known competing financial interests or personal relationships that could have appeared to influence the work reported in this paper.
